# The effects of oral administration of *Cola nitida* on the pharmacokinetic profile of metoclopramide in rabbits

**DOI:** 10.1186/s40360-019-0379-6

**Published:** 2020-01-06

**Authors:** Cecilia Nwadiuto Amadi, Wisdom Izuchukwu Nwachukwu

**Affiliations:** Department of Experimental Pharmacology & Toxicology, Faculty of Pharmacy, University of Port Harcourt, PMB 5323, Choba, Port Harcourt, Rivers State Nigeria

**Keywords:** Metoclopramide (MCP), *Cola nitida*, Herb-drug interaction, P-glycoprotein, Metabolizing enzymes

## Abstract

**Background:**

*Cola nitida* is commonly chewed in many West African cultures to ease hunger pangs and sometimes for their stimulant and euphoriant qualities. Metoclopramide is a known substrate for P-gp, SULT2A1 and CYP2D6 and studies have revealed that caffeine- a major component of *Cola nitida* can induce P-glycoprotein (P-gp), SULT2A1 and SULT1A1, hence a possible drug interaction may occur on co-administration. The aim of this study was to investigate the pharmacokinetic interactions of *Cola nitida* and metoclopramide in rabbits.

**Methods:**

The study was performed in two stages using five healthy male rabbits with a 1-week washout period between treatments. Stage one involved oral administration of metoclopramide (0.5 mg/kg) alone while in the second stage, metoclopramide (0.5 mg/kg) was administered concurrently with *Cola nitida* (0.7 mg/kg). Blood samples were collected after each stage at predetermined intervals and analyzed for plasma metoclopramide concentration using HPLC.

**Results:**

Compared with control, the metoclopramide/*Cola nitida* co-administration produced a decrease in plasma concentration of metoclopramide at all the time intervals except at the 7th hour. The following pharmacokinetic parameters were also decreased: area under the curve (51%), peak plasma concentration (39%), half-life (51%); while an increase in elimination rate constant (113%) and clearance rate (98%) were noted indicating rapid elimination of the drug. A minimal decrease in absorption rate (10%) was also observed.

**Conclusions:**

The results of this study reveal a possible herb-drug interaction between *Cola nitida* and metoclopramide.

## Background

Herbs have been used for many decades to improve general health and wellbeing and are frequently considered “natural” and “safe” [1]. However, herbs contain biologically active components that can potentially interact with drugs. Studies have revealed that phytochemical constituents of botanicals interfere with certain enzymes and transporters, hence affecting the way substrate drugs are absorbed and metabolized [1]. For example, furanocoumarins found in grapefruit irreversibly bind with CYP3A4, leading to immense increase in the absorption of simvastatin [2]. In addition, grapefruit juice has also been reported to increase the blood levels of cyclosporine by 38%, tacrolimus by 110%, and oxycodone by 67% respectively [3].

Kola nut is a tropical herb chewed in many West African cultures individually or in a group setting [4]. Usually, it is presented ceremonially to tribal chiefs or to guests during important celebrations or occasions [4]. Kola nuts are consumed mainly for their stimulant and euphoric properties and also used to ease hunger pangs. They exhibit similar effects as other xanthine-containing herbs such as cocoa, tea, etc. [5]. Kola nut extract is gotten from the seeds of majorly two tropical Cola species (*Cola nitida* or *Cola acuminata)* of the Family, Sterculiaceae [6]. The extract from kola nut is used in the food industry as a flavoring agent. Daily consumption rate of kola nut extract is 0.69 mg/kg/day [6]. The major constituents of kola nut are purine alkaloids such as caffeine and theobromine [6]; flavonoids such as Epicatechin and catechin [7]. Other phytochemical constituents include saponins, cardenolides and tannins [4].

P-glycoprotein (P-gp), a major efflux pump for lipophilic and cationic drugs, and cytochrome P450 enzymes (CYP450s) have been demonstrated to play important roles in the oral bioavailability of many drugs and drug-drug interactions [8, 9]. Green tea catechins have been demonstrated to influence the activities of drug transporters such as P-glycoprotein and organic anion transporting polypeptides in vitro [10]. Studies by Misaka et al. [11] to probe the effects of the oral administration of (−)-epigallocatechin-3-gallate (EGCG) and green tea extract (GTE) on pharmacokinetic properties of nadolol, revealed a significant decrease in the maximum plasma concentration (C_max_) and area under the concentration curve (AUC) of nadolol by 85% and 74%, respectively.

Metoclopramide is a prokinetic drug which is due to its 5-HT_4_ receptor agonist effect. It is commonly used to treat gastroparesis, a chronic stomach motility disorder characterized by delayed gastric emptying in the absence of mechanical obstruction [12]. Metoclopramide is also frequently used as an antiemetic drug. It exhibits linear pharmacokinetics with an elimination half-life of 5 to 6 h [12]. The absorption of metoclopramide in the small intestine is majorly mediated by P-gp [13–15]. Metabolism of metoclopramide is mainly via conjugation to sulfated and glucuronidated metabolites, although oxidative metabolism by CYP2D6 has also been reported [15–18]. In addition, studies by Senggunprai et al. [19] revealed that SULT2A1 plays an important role in metoclopramide metabolism. Another study by Zhou et al. [20], demonstrated that caffeine significantly induced SULT2A1 and SULT1A1 in rat intestine and liver suggesting that consumption of caffeine/caffeine-containing diets can induce drug - metabolizing SULTs in tissues involved in drug detoxification. Furthermore, caffeine is reported to be an inducer of the enzyme P-glycoprotein (P-gp) [21]. The summation of the effects of caffeine (a major constituent of *Cola nitida*) on these enzymes could result in reduced absorption and increased drug metabolism rate, which could subsequently cause therapeutic failure and drug resistance. It is reasonable to suspect that caffeine might alter the pharmacokinetics of various substrates of P-gp, SULT2A1, SULT1A1, and CYPs. In view of this, the potential drug–drug interaction of metoclopramide mediated by SULT2A1, CYP450, and P-gp might occur. Consequently, in the present study, we evaluated the effect of administration of *Cola nitida* on the pharmacokinetics of metoclopramide in rabbits.

## Methods

### Materials and reagents

Methylated spirit (JHD, China), Sodium hydroxide, Sulphuric acid (99%) and acetonitrile were obtained from Loba Chemie Pvt. Ltd., metoclopramide tablets USP (10 mg, TEVA UK Limited, Eastbourne). All other chemicals were of HPLC grade.

### Sample identification and processing

*Cola nitida* seeds were obtained from Choba Market in Port Harcourt, Nigeria in July 2018 and authenticated by Dr. M. Suleiman, Pharmacognosy department, Faculty of pharmaceutical Sciences, University of Port Harcourt. A voucher specimen (Ref. No. UPHM 307) was deposited at the university herbarium. The *Cola nitida* seeds were dried at room temperature and pulverized with a blender. A total of 8. 2 g of the powdered *Cola nitida* was suspended in 100 mL of 2% v/v Tween 80 to make an 82 mg/mL suspension.

### Drug administration and blood collection

Five New Zealand strains of adult male rabbits (1.88 ± 0.7 kg) used for the study were obtained from the animal house of the department of Experimental Pharmacology and Toxicology, Faculty of Pharmaceutical Sciences, University of Port Harcourt. Animals were handled in accordance with international guidelines and experimental procedures followed the approved guideline of the Ethical Committee on Animal Studies of the university of Port Harcourt (Approval number UPH/PHARM/2017/046). The animals were acclimatized for two weeks with free access to food and water before the experiment. Animals were fasted for 12 h prior to dosing and fed approximately 4 h post-dose. Experiment was carried out in two stages with a 1-week drug washout period between treatments according to the method previously described by Nwafor et al. [22]. Stage one involved oral administration of metoclopramide (0.5 mg/kg) [23] alone while in the second stage, metoclopramide (0.5 mg/kg) was administered concurrently with *Cola nitida* (0.7 mg/kg). A dose of 0.7 mg/kg of *Cola nitida* comparable to human dose was chosen for the second phase of this study [6]. Blood samples were collected at 0.5, 1, 2, 4, 5.5, and 7 h after dosing from the marginal ear vein of the rabbits, centrifuged for 20 min at 4000 rpm and stored in a freezer until HPLC analysis. At every stage of blood withdrawal, the ear was cleaned with 95% v/v alcohol and local anaesthetic cream applied on the collection site 10 min prior to sampling. The results obtained were plotted as mean metoclopramide plasma concentration versus time to obtain a plasmatic curve, from which the pharmacokinetic parameters were calculated. On completion of studies, all animals were returned to the animal house of the department of experimental pharmacology and toxicology, University of Port Harcourt, Nigeria.

### Metoclopramide assay

The plasma concentrations of metoclopramide were assayed using HPLC with UV absorbance detection as reported by Cossu et al. [23]. Liquid-liquid extraction was done using Methylene chloride as solvent. Following centrifugation, the organic phase was evaporated and the residue reconstituted with the mobile phase prior to HPLC analysis. The resultant turbid solution was centrifuged and the supernatant analyzed using HPLC with UV absorbance (Rayleigh model LT 100) to ascertain the amount of metoclopramide extracted. ODS Waters XTerra™ column (250 × 4.6 mm, 5 μm particle size, Waters, Milan, Italy) was used for sample separation and quantification at room temperature. A volume of 100 μL samples were injected and elution was done with mixtures of acetonitrile and 0.01 N sulphuric acid 15/85 (v/v) (the mobile phase) at a flow rate of 1.5 mL/min. The UV detection wavelength was 213 nm wavelength. The calibration curve for metoclopramide was linear over the range of 0–100 ng/mL.

### Pharmacokinetic evaluation

The pharmacokinetic parameters of metoclopramide were determined using the non-compartmental method. The elimination half-life (T_1/2_) was obtained using equation: 0.693/K_el_. Log-linear regression was used to determine the elimination rate constant (K_el_). The area under the curve (AUC) was determined from 0 to 7 h [AUC (0–7)] using the trapezoidal method. AUC from time zero (0) to infinity [AUC (0-∞)] was calculated as AUC (0–7) plus AUC from 7 h to infinity [AUC (7- ∞)]. The peak plasma concentration (C_max_) and the time to attain maximum plasma concentration _(_T_max_) were obtained directly from the experimental data. The back-feathering method was used to obtain the absorption rate constant (K_a_) from the absorption phase with the relationship: slope = − 0.43K_a_. Clearance rate (CL_T_) of metoclopramide from plasma was calculated using: CL_T_ = K_el_.V_d_; where V_d_ is the volume of distribution.

### Statistical analysis

Results were expressed as mean ± standard deviation. The pharmacokinetic parameters were compared with a one-way analysis of variance (ANOVA), followed by the Student’s *t*-test for comparison between control and the test groups. A *P* value of < 0.05 was considered statistically significant.

## Results

**Fig. 1:**
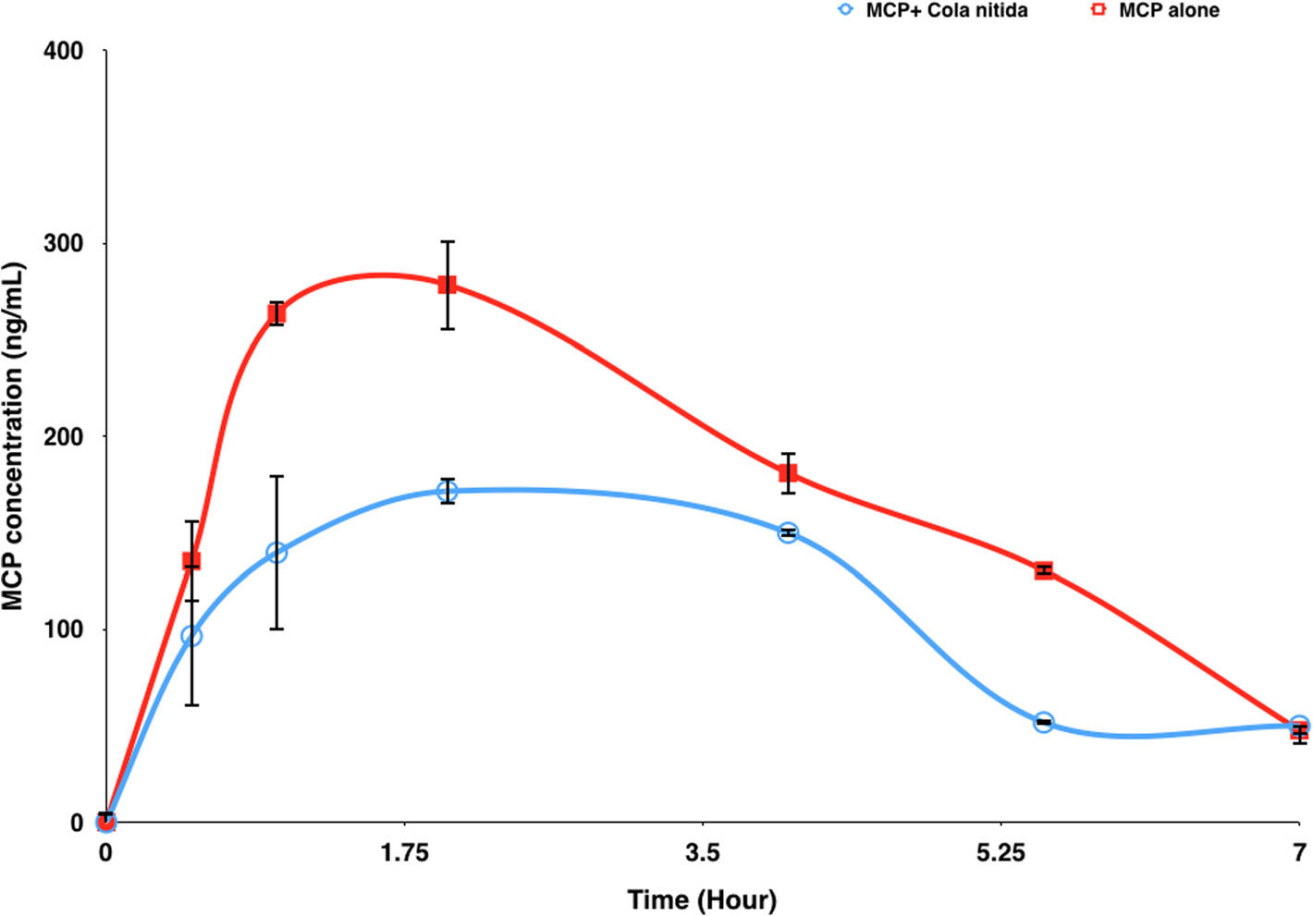
Mean plasma concentration-time profiles of metoclopramide after an oral administration of metoclopramide (0.5 mg/kg) to rabbits in the presence and absence of *Cola nitida* (mean ± STD, *n* = 5). MCP (metoclopramide, 0.5 mg/kg, p.o.); MCP + *Cola nitida* (combined use with 0.7 mg/kg of *Cola nitida*)

**Fig. 2:**
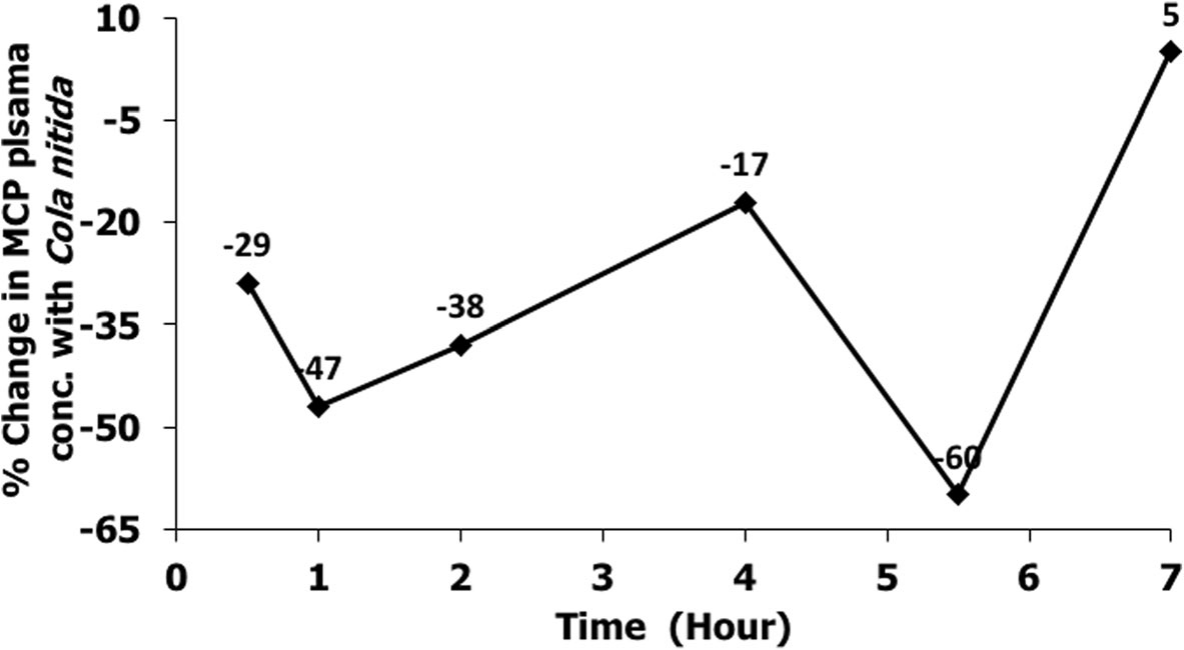
Percentage change in metoclopramide (MCP) plasma concentration (0–7 h) when co-administered with *Cola nitida*

The plasma concentration-time profiles and the mean pharmacokinetic parameters following an oral administration of metoclopramide (0.5 mg/kg) in the presence and absence of *Cola nitida* (0.7 mg/kg) are shown in Fig. 1 and Table 1 respectively. The plasma concentration of metoclopramide was quantified up to 7-h post dose. From Fig. 1 and Table 1, co-administration of *Cola nitida* with metoclopramide in rabbits caused a decrease in the plasma concentration of metoclopramide compared to the group that received metoclopramide alone (control group). Results from the group that received *Cola nitida* and metoclopramide concurrently revealed a decrease in AUC (0-7 h) and C_max_ by 51% and 39% respectively. The increase in AUC was significant at *P* < 0.05. Elimination rate and clearance rate increased by 113% and 98% respectively, while absorption rate constant decreased by 10% resulting in lowered plasma concentrations of metoclopramide. The time to attain the maximum plasma concentration (T_max_) was delayed in the presence of *Cola nitida* although there was no statistical significance (*P* < 0.05). T_max_ increased by 33% (from 1.5 h to 2 h) while the half-life of metoclopramide decreased by 51% from (4.5 h to 2.2 h) in the group that received *Cola nitida* compared to the control group*.* The decrease in half-life was significant at *P* < 0.05. The percentage change in metoclopramide concentration (0.5–7 h) due to *Cola nitida* administration is illustrated in Fig. 2. There was a 29% decrease in plasma concentration of metoclopramide at 0.5 h in the group that received *Cola nitida.* At 1, 2, 4 and 5.5 h, there were 47%, 38%, 17% and 60% decrease respectively. However, at 7 h, there was a 5% increase compared to control suggestive of reduced elimination rate.

**Table 1:** Pharmacokinetic parameters of metoclopramide after an oral administration of metoclopramide (0.5 mg/kg) to rabbits in the presence and absence of *Cola nitida* (mean ± SEM, *n* = 5). *P < 0.05, compared to the control given metoclopramide alone. T_max_ (hr) = time to reach peak blood concentration; C_max_ (ng/mL) = peak blood concentration; AUC0-7 h (ng/h/mL) = area under the blood concentration time curve; K_a_ (hr-1) = absorption rate constant; CL (L/hr./kg) = clearance rate; T_1/2_ = Half-life; K_el_ = elimination rate constant

	AUC0-7 h ng/h/mL)	Tmax hr)	Cmax (ng/mL)	Ka (hr-1)	CL L/hr./kg)	T ½ hr)	Kel (hr-1)
MCP	1688.2 ± 112.2	1.5 ± 0.03	280 ± 15.30	0.80 ± 0.01	0.56 ± 0.01	4.5 ± 0.05	0.15 ± 0.02
MCP + K	821.06 ± 61.4*	2+ 0.03	171.49 ± 23.50	0. 72 ± 0.05	1.11 ± 0.03	2.2 ± 0.05*	0.32 ± 0.02
% Change from control	−51	33	−39	−10	98	−51	113

## Discussion

This study was done using an experimental design previously described by Nwafor et al. [22]. Sample size (*n* = 5) was used and experiments were carried out in two stages, with the first stage serving as control. An improvement to this design would be to increase sample size and to randomize the animals.

The results obtained from our study demonstrated that the co-administration of metoclopramide with *Cola nitida* significantly decreased both the peak blood concentration and systemic exposure of metoclopramide, indicating that the oral bioavailability of metoclopramide was reduced by *Cola nitida*. Clearance (CL), which is the volume of blood cleared of drug per unit time [24], increased by 98% when *Cola nitida* was co-administered with the metoclopramide. The increase in the CL means that more of the metoclopramide will be cleared from the body at any given time when administered with *Cola nitida*. The K_el_ of metoclopramide increased by 113% with *Cola nitida* administration. K_el_ reflects the rate at which drug is removed from the body, it implies that the *Cola nitida* enhanced the rate of metoclopramide elimination resulting to reduced plasma concentration. Metoclopramide enhances gastric emptying, therefore, the increased Tmax of oral metoclopramide in the presence of *Cola nitida* may be explained by the delayed gastric emptying due to reduced pharmacological activity of the drug caused but the herb. The absorption rate constant (Ka) increased minimally by 7% suggesting a weak inhibition of P-gp-mediated efflux.

Studies have shown that P-gp, SULT2A1, and CYP2D6 play key roles in the oral bioavailability of metoclopramide [13–19]. Therefore, we hypothesize that the efflux activities of P-gp in the intestine and/or the catalytic functions of CYP2D6 and SULT2A1 in the liver and/or intestine were possibly activated by *Cola nitida* which produced a decreased bioavailability of metoclopramide in rabbits.

Flavonoids (such as epicatechin and catechin), furanocoumarins and alkaloids (such as caffeine) found in fruits, vegetables and herbs have been reported to modulate the activities of drug transporters such as P-glycoprotein and metabolizing enzymes [10, 25]. For example, green tea extract and/or (−)-epigallocatechin-3-gallate was demonstrated to reduce the bioavailability of quetiapine, sunitinib, clozapine, and nadolol by influencing the activities of drug-metabolizing enzymes and drug transporters [26]. Phytochemical evaluation of *Cola nitida* has indicated the presence of purine alkaloids such as caffeine and theobromine [6]; flavonoids such as epicatechin and catechin [7] in addition to saponins, cardenolides and tannins [4].

Given that metoclopramide is a substrate for P-gp, SULT2A1 and CYP2D6, the reduced oral exposure/bioavailability of metoclopramide may have resulted from the stimulatory effect of *Cola nitida* on metabolism, increased hepatic elimination of metoclopramide in addition to the decreased intestinal absorption via the weak stimulation of P-gp- mediated drug efflux. The findings from our study are suggestive of possible mechanisms not investigated in the present work.

The study established significant alteration in the pharmacokinetic indices of metoclopramide caused by *Cola nitida* co-administration which could result in therapeutic failure. Our data suggest that the combined use of *Cola nitida* or caffeine-containing supplements with metoclopramide may require close monitoring for potential drug-herb interaction.

## Conclusions

*Cola nitida* produced a reduction in the oral exposure/bioavailability of metoclopramide. This reduction strongly suggests an increased catalytic activity of CYP2D6 and SULT2A1 and/or efflux function of P-gp in the intestine and/or liver by *Cola nitida*. These could be potential mechanisms that were not investigated in the present work necessitating further studies for validation. The results of our study reveal a possible herb-drug interaction involving *Cola nitida* and metoclopramide suggesting that a concomitant use of *Cola nitida* or cola-containing diet may require close monitoring.

## Data Availability

The datasets used and/or analysed during the current study are available from corresponding author on reasonable request.

## Abbreviations

ANOVA: Analysis of variance
AUC: Area Under the blood Concentration- time curve
CL: Clearance
C_max_: Peak blood concentration
CYP: Cytochrome P450 enzyme
HPLC: High Performance Liquid Chromatography
K_a_: Absorption rate constant
K_el_: Elimination rate constant
P-gp: P-glycoprotein
STD: Standard deviation
SULT1A1: Sulfotransferase 1A1
SULT2A1: Sulfotransferase 2A1
T_1/2_: Half-life
T_max_: Time to reach peak blood concentration
